# Cryotherapy is Associated with Improved Clinical Outcomes of Sorafenib Therapy for Advanced Hepatocellular Carcinoma

**DOI:** 10.1007/s12013-012-9353-2

**Published:** 2012-04-05

**Authors:** Yongping Yang, Yinying Lu, Chunping Wang, Wenlin Bai, Jianhui Qu, Yan Chen, Xiujuan Chang, Linjing An, Lin Zhou, Zhen Zeng, Min Lou, Jiyun Lv

**Affiliations:** 1 Center of Therapeutic Research for Hepatocellular Carcinoma, Beijing the 302nd Hospital, 100 Xisihuan Middle Road, Beijing, 100039 China; 2 Infectious Diseases Institution of Beijing, Beijing, 100039 China

**Keywords:** Hepatocellular carcinoma, Sorafenib, Cryotherapy, Microvessel density, Efficacy, Safety

## Abstract

We assessed the safety and efficacy of sorafenib with cryotherapy (cryoRx) in advanced hepatocellular carcinoma (HCC). One hundred four HCC patients were enrolled, who met the following criteria: (i) Barcelona Clinic Liver Cancer stage C; (ii) HCC without distant metastasis; (iii) the presence of portal vein thrombosis (PVT); (iv) Child-Pugh class A or B; and (v) life expectancy of at least 12 weeks. The patients were randomly divided into sorafenib-cryoRx and sorafenib (control) groups. Primary endpoint was time to progression (TTP); secondary endpoints included overall survival (OS) and tolerability. Microvessel density (MVD) was assessed by CD34-immunostaining. After a median 10.5 (4–26) months follow-up, the data showed that median TTP was 9.5 (8.4–13.5) months in combinatorial therapy group vs. 5.3 (3.8–6.9) months in sorafenib group (*P* = 0.02). The median OS was 12.5 (95 % CI 10.6–16.4) months in combination therapy group vs. 8.6 (7.3–10.4) months in sorafenib group (*P* = 0.01). Low MVD patients in combination therapy exhibited significantly longer median TTP and OS than controls. High MVD was predictive of poor responses to sorafenib. CryoRx did not increase frequency/degree of sorafenib-related adverse events. Therefore, it was concluded that the addition of cryoRx significantly improved clinical outcomes of Sorafenib therapy in advanced HCC with acceptable tolerance and similar safety profiles as previously reported.

## Introduction

Hepatocellular carcinoma (HCC) is the third most common cause of cancer-related deaths worldwide [1] and the second most common cause in China [2]. Most patients with advanced HCC at the time of initial diagnosis exhibit poor outcomes [3]. In China, HCC is most commonly caused by infection with the hepatitis B virus (HBV) [4]. The incidence of HCC has increased in recent years, largely because of chronic HBV infection-related liver cirrhosis [5]. Therapeutic options are stage-dependent [6, 7]. Only about 30 % of patients who presented with early-stage tumors undergo resection, liver transplantation, and percutaneous ablation due to various factors, such as multifocal tumor and poor liver function resulting from underlying cirrhosis [8–10]. For a long period, no effective treatment was available for these conditions [11]. Sorafenib is a newly developed, molecularly targeted agent. This multikinase inhibitor demonstrated significant survival benefits in phase III trials for patients with advanced HCC [12, 13]. However, its efficacy remains moderate, and some patients still show a very short period of survival following treatment [14].

The mechanisms causing some patients to become refractory to sorafenib are currently unclear. High intratumoral microvessel density (MVD) was associated with higher level of VEGF/VEGHFR signaling pathway activity. As such, the presence of high MVD in advanced HCC patients may be associated with a positive response to sorafenib treatment. It is currently unknown, however, whether the presence of high intratumoral MVD can affect responses to sorafenib treatment in advanced HCC patients. Several previous findings suggest a strong rationale for combining both treatment modalities. In mice with implanted renal tumors, the combination of radiofrequency ablation and sorafenib was found to cause an increase in the efficacy of tumor ablation that was dependent on sorafenib dosage [15]. Cryotherapy (cryoRx), based on in situ freezing and devitalization of tissues, has been found more advantageous than surgical resection in cirrhotic patients because its focal application results in the loss of less hepatic parenchyma. Moreover, it is possible to treat several liver segments and the technique can be applied and controlled precisely to produce a predictable zone of necrosis. This technology has been used extensively in open surgical settings and, more recently, applied percutaneously to treat renal tumors and liver metastases [16, 17]. Therefore, the aim of the current study was to confirm the efficacy and safety of combined application of sorafenib and cryotherapy in advanced HCC, and to also study the ablation tumor burden impact for sorafenib therapy responses.

## Materials and Methods

### Patients’ Classification and Eligibility Criteria

Based on the Barcelona Clinic Liver Cancer (BCLC) staging classification [7], 296 consecutive patients with HBV-related advanced HCC were screened between July 2008 and July 2010 at the Center of Therapeutic Research for Hepatocellular Carcinoma, Beijing the 302nd Hospital, China. Fifty-seven patients were classified as Child-Pugh C, 38 patients with Child-Pugh B8 or B9 and their serum bilirubin mean level was >51.3 μmol/L. Twenty three patients had life expectancy of <12 weeks. Ten patients had Eastern Cooperative Oncology Group Performance Status (ECOG PS) ≥3, and 64 patients with a history of hepatectomy (8), preoperative chemotherapy (6), prior transarterial chemoembolization (TACE) or local ablation (44), and radiotherapy (6). As a result, 192 patients were excluded from this study. Thus, a total of 104 patients with advanced HCC were eligible for this study (Table 1). The diagnosis of HCC [6] was indicated by imaging findings and confirmed by biopsy (single action biopsy device, 16 g; Promex Technologies, USA). PVT, as a sign of macroscopic vascular invasion and extrahepatic spread, was used to define advanced HCC, but patients exhibiting extrahepatic spread were excluded from the study. Eligibility criteria also included the ECOG PS of 0, 1, or 2; Child-Pugh class A or B; life expectancy of at least 12 weeks; total bilirubin concentration of ≤51.3 μmol/L; and HBsAg positive status. In addition, the patients considered for inclusion were required to exhibit at least one tumor lesion that could be measured along one dimension according to modified Response Evaluation Criteria in Solid Tumors (mRECIST) assessment for HCC [18].

**Table 1:** Demographic and baseline characteristics of patients

Characteristic	Combination therapy (*N* = 52)	Sorafenib (*N* = 52)
Age (years)	51.2 ± 11.9	52.6 ± 8.3
Gender (no; %)		
Male	48 (92.3)	47 (90.4)
Female	4 (7.7)	5 (9.6)
ECOG performance status (no; %)		
0	16 (30.8)	17 (32.7)
1	29 (55.7)	30 (58)
2	7 (13.5)	5 (9)
BCLC stage C (no; %)	52 (100)	52 (100)
Tumor diameter (cm; range)	8.39 ± 4.38 (3.5–12.8)	8.32 ± 2.72 (3.2–13.2)
Number of tumor sites (no; %)		
1	7 (13.5)	6 (11.5)
2	9 (17.3)	10 (19.2)
3	10 (19.2)	11 (21.2)
≥4	26 (50)	25 (48.1)
Macroscopic vascular invasion (no; %)		
Branch	36 (69.3)	37 (71.2)
Trunk	16 (30.7)	15 (28.8)
Tumor differentiation (no; %)		
Well	9 (17.3)	10 (19.2)
Intermediate	30 (57.7)	30 (57.7)
Poorly	13 (25)	12 (23.1)
HBV-DNA positivity (no; %)		
100–9,999	22 (42.3)	24 (46.2)
10,000–99,999	19 (36.5)	18 (34.6)
≥100,000	11 (21.2)	10 (19.2)
Child-Pugh class (no; %)		
A	41 (78.8)	43 (83)
B	11 (21.2)	9 (17)

*HBV* Hepatitis B virus, *ECOG* Eastern Cooperative Oncology Group

### Study Design

According to Sorafenib HCC Assessment Randomized Protocol (SHARP) trial [13], the overall survival (OS) rate of the advanced HCC patients for sorafenib at the 15th month was 37 %. The sample size calculation was based on the detection of significant differences in OS, the second endpoint parameter of this trial, assuming that OS rate was 50 % for the combination therapy group at the 15th month. A total of 90 patients were required for a log-rank test with an overall two-sided significance level of 0.05 and power of 0.805. From our experience, it can be expected that 15–20 % of the patients will drop out after randomization. In order to accommodate for the drop-out rate, the total sample size was, therefore, increased to 104. The study was investigator initiated and was approved by the institutional ethics committee. Written informed consent was obtained from the patients before enrollment. All the eligible patients were randomly assigned, with a 1:1 ratio, to either sorafenib + cryoRx group (*N* = 52) or sorafenib-alone group (*N* = 52) using simple randomization by means of computer to achieve a balance between the two groups. None of the patients had prior treatment, such as chemotherapy or radiation therapy.

### Sorafenib Administration

All the patients received sorafenib at a dose rate of 400 mg twice daily for at least 8 weeks. Treatment interruptions and dose reductions (first to 400 mg twice daily, and then to 200 mg twice daily) were permitted in case of adverse drug reactions (ADRs) according to the National Cancer Institute Common Toxicity Criteria [19]. For ADRs of grades 3–4, sorafenib was withdrawn to 200 mg twice daily until the ADRs improved to grade ≤2, and then increased to 400 mg twice daily if well tolerated. The discontinuation of therapy met the following criteria: ADRs that required termination of medication, deterioration of ECOG PS score to 4, and withdrawal of consent. If disease progression was observed, then sorafenib was continued when the patient was considered to have a good clinical status (e.g., PS, liver function and tolerable side effects) and wished to continue the treatment. Following sorafenib treatment, cryoRx were conducted in those without absolute contraindications, based on the potential clinical benefits expected from the treatment and the patient’s consent. Sorafenib therapy was continued without interruption during local therapies.

### Cryotherapy Procedure

Argon–helium cryoablation was performed as we described elsewhere [20]. In brief, the size and number of probes depended on the location and the average size of the lesions to be ablated. An argon–helium gas-based CryoCare system (EndoCare, Inc., CA, USA) and cryoprobes (2 and 3 mm) were used to freeze the tumor with a dual freeze–thaw cycle under ultrasound-guidance. Based on patient’s ECOG PS, liver function and tolerable side effects, tumor burden, or new recurrence was best cryoablated in four times at most.

### CD34 Immunohistochemical Staining

All the samples from the HCC patients were reviewed histologically using hematoxylin and eosin (H&E) staining; the paraffin-embedded samples were cut into 5-μm-thick sections and processed for immunohistochemistry according to the manufacturer’s instructions and as previously described [21, 22]. Tumorous sections were immunostained with human CD34 monoclonal antibody (BioGenex, San Ramon, CA, USA). The tissue sections were incubated with primary CD34 monoclonal antibody (BioGenex, CA, USA) diluted 1:50 with Tris-buffered saline solution for 60 min at room temperature. Afterward, as secondary-biotinylated anti-mouse immunoglobulin antibody (Dako, USA) was used at a concentration of 1.0 μg/mL and allowed to react for 30 min at 37 °C. Then, streptavidin-biotinylated horseradish peroxidase complex (Dako, USA) was added. The negative control was obtained by substituting primary antibodies with mouse immunoglobulin G (IgG).

### Determination of Microvessel Density

The intratumoral MVD was evaluated by two independent observers who were blinded to the patients’ clinical data. The tissue sections were screened at a low power field (×40) and five areas with the most intense neovascularization (hot spots) were selected. Microvessel counts of these areas were performed under a high power field (×200). To reduce observer-related variation, counting of microvessels was performed using computer image analyzer (MetaMorph Imaging System Version 3.0; Universal Imaging Corp, West Chester, PA, USA). Microvessels, tumor cells, and connective elements were counted as one microvessel, irrespective of the presence of a vessel lumen. The mean microvessel count of the five most vascular areas was taken to constitute the MVD which was expressed as the absolute number of microvessels per 0.74 mm^2^ (at ×200 magnification).

### Disease Assessment

The disease assessment was performed by computed tomography (CT) scan or magnetic resonance imaging (MRI), approximately every 8 weeks. Response was determined by independent radiologists and classified according to mRECIST assessment for HCC [18]. In this study, responses were classified into complete response (CR), partial response (PR), stable disease (SD), or progressive disease (PD). Patients who achieved CR, PR, or SD were defined as achieving clinical benefits (CB). Patients who exhibited CR or PR were defined as achieving a clinical efficacy response (CER).

### End Points

The primary endpoint of the study was time to progression (TTP). The secondary endpoints included OS, the disease-control rate (DCR), and tolerability. TTP was calculated from the date of commencement of sorafenib to the date of disease progression or death. OS was calculated from the date of commencement of sorafenib to the date of death or the last follow-up.

### Statistical Analysis

The data were analyzed using SPSS13 statistical software package (SPSS, Chicago, IL, USA) and expressed as median and range values. All continuous data were classified into subgroups according to the median for analysis. Associations between OS, TTP, and potential prognostic factors were assessed by the Kaplan–Meier method (log-rank test) in a univariate analysis. All *P*-values <0.05 were considered statistically significant.

## Results

### Patients’ Characteristics

The combination therapy and sorafenib alone groups were well balanced with regard to baseline demographic and disease characteristics (Table 1). Eighty-four (80.8 %) patients were classified as Child-Pugh class A, and 20 (19.2 %) patients were Child-Pugh class B. Thirty-three (31.7 %) patients were ECOG PS 0, 59 (56.7 %) were ECOG PS 1, and 12 (11.6 %) were ECOG PS 2. The tumor differentiation was well in 19 (18.3 %) patients, intermediate in 60 (57.7 %), and poorly in 25 (24.0 %). The HBV DNA loads were low in 46 (44.2 %) patients, moderate in 37 (35.6 %), and high in 21 (20.2 %).

### Adverse Events

With regard to non-hematologic toxicity, rash was observed most commonly (62 %), followed by hypertension (56 %), weight loss (52.9 %), alopecia (50 %), diarrhea (46 %), fatigue (43.3 %), hand-foot skin reaction (HFSR; 42 %), liver dysfunction (34.6 %), voice change (18 %), abdominal pain (12.5 %), and upper gastrointestinal tract bleeding (16 %). Moreover, grade 3 or 4 non-hematologic toxicities included HFSR, diarrhea, liver dysfunction, and upper gastrointestinal tract bleeding which occurred in 12.2, 12, 6.4, and 6 % of patients, respectively. With respect to hematologic toxicity, leukopenia was the most common sign of toxicity (24 %), followed by thrombocytopenia (12 %) and anemia (8 %). All four patients with anemia exhibited grade 3 or 4 toxicity, and one patient’s hemoglobin was reduced to 32 g/L.

### Overall Response and Efficacy

**Fig. 1:**
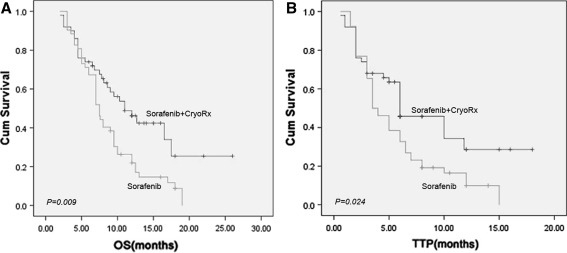
Kaplan–Meier estimates of OS and TTP.** a** Kaplan–Meier survival curves are shown for 52 patients treated with combination therapy and 52 patients treated with sorafenib alone. Median OS was significantly longer (*P* = 0.009) in patients from combination therapy group than in patients from sorafenib alone group;** b** Kaplan–Meier survival curves showing significantly longer TTP (*P* = 0.04) in patients from combination therapy group than in patients from sorafenib alone group

The median follow-up time was 10.5 (range 4.0–26.0) months, and the median duration of sorafenib treatment was 7.5 (2.5–26.0) months. Ten (9.6 %) patients discontinued sorafenib at 6–24 weeks on account of liver function deterioration (6 cases) and esophagogastric varices bleeding (4 cases), 21 (20.2 %) patients received the reduced sorafenib dosage (200 mg twice daily) because of grades 3–4 ADRs, but all these patients restored to 400 mg twice daily dose after 1–2 weeks. Overall, the patients receiving combination therapy had a median OS of 12.5 (95 % CI 10.6–16.4) months, compared with 8.6 (95 % CI 7.3–10.4) months for those receiving sorafenib alone (log-rank *P* = 0.009; Fig. 1a). In addition, the patients in combination therapy group had a significantly longer median TTP (9.5 months; 95 % CI 8.4–13.5 months) than the patients in sorafenib alone (5.3 months; 95 % CI 3.8–6.9 months) group (log-rank *P* = 0.024; Fig. 1b). Regarding the analysis for best response, 4 of 52 (7.6 %) patients in combination therapy exhibited CR, 9 (17.3 %) patients exhibited PR, 22 (42.3 %) patients exhibited SD, whereas in the sorafenib 4 (7.6 %) and 19 (36.5 %) patients exhibited PR and SD, respectively. The rates of CER and DCR (Table 2) were significantly higher for combination therapy (CER 22 % and DCR 66 %) than those for sorafenib alone (CER 9.6 %; *P* = 0.0414 and DCR 46.2 %; *P* = 0.0272). Both combination therapy and sorafenib alone exhibited a clinical benefit in all preplanned subgroup analyses, despite some patients having characteristics associated with poor prognosis including poorer ECOG PS, tumor diameter >7 cm, high HBV DNA load, Child-Pugh class B, fatigue, weight-loss, abdominal pain, and liver dysfunction (Table 3). Disease progression occurred in 86 (82.6 %) patients. Furthermore, 53 (50.9 %) patients died that included: 25 (24.0 %) due to recurrence/metastasis, 13 (12.5 %) due to liver failure, 8 (7.7 %) due to esophagogastric varices bleeding, 4 (3.8 %) due to refractory ascites-induced renal failure, and 3 (2.9 %) due to tumor rupture/hemorrhage.

**Table 2:** Summary of efficacy measures

Outcome	Combination therapy (*n* = 52)	Sorafenib (*n* = 52)	*P*-value
Over survival (months)			0.009
Median	12.5	8.6	
95 % CI	10.6–16.4	7.3–10.4	
TTP (months)			0.024
Median	9.5	5.3	
95 % CI	8.4–13.5	3.8–6.9	
Level of response (no; %)			
Complete response	4 (7.6)	0	NA
Partial response	9 (17.3)	5 (9.6)	0.1186
Stable disease	22 (42.3)	19 (36.5)	0.4423
Clinical efficacy rate (no; %)	13 (25.0)	5 (9.6)	0.0414
Disease control rate (no; %)	35 (67.3)	24 (46.2)	0.0272

*Clinical efficacy rate* It was defined as the proportion of patients who had the best response rating of complete response or partial response which was maintained for ≥4 weeks from the first manifestation of that rating

*Disease control rate* It was defined as the proportion of patients who had the best response rating of either complete/partial response or stable disease which was maintained for ≥4 weeks from the first manifestation of that rating

**Table 3:** Univariate analysis of patients’ demographic and clinical characteristics for predictive factors of DCR, TTP and OS

Parameter	No. of patients				*P*-value of DCR	TTP (months)		OS (months)	
	Total	DCR	PD	Died		Median	*P*	Median	*P*
Sex					1.000		0.514		0.781
Male	95	54	41	48		6.0		10.5	
Female	9	4	5	5		4.5		9.0	
Age					0.526		0.668		0.228
≤51	52	28	24	28		5.0		9.0	
>51	52	30	22	25		6.0		10.5	
ECOG PS					<0.001		<0.001		<0.001
0	33	23	10	10		8.5		17.0	
1	59	31	28	35		6.0		11.0	
2	12	4	8	8		3.0		6.5	
Tumor differentiation					0.473		0.155		0.401
Well	19	11	8	10		4.0		8.0	
Intermediate	60	34	26	29		4.5		9.0	
Poorly	25	13	12	14		3.5		7.0	
Tumor diameter (cm)					0.034		0.025		0.007
≤7	52	38	17	19		6.5		12.0	
>7	50	20	29	34		4.0		8.1	
Tumor number					0.012		0.165		0.995
1	13	11	2	3		6.0		12.7	
2	19	13	6	7		6.0		11.0	
3	21	15	6	12		5.0		10.0	
4	51	19	32	31		4.0		10.0	
HBV DNA (IU/mL)					0.001		<0.001		<0.001
0–9,999	46	30	16	17		6.0		12.7	
10,000–99,999	37	20	17	21		4.0		10.0	
≥100,000	21	8	13	15		3.0		8.0	
Child-Pugh class					0.027		<0.001		0.004
A	84	51	33	39		6.0		9.5	
B	20	7	13	14		3.5		5.0	
Fatigue					0.002		<0.001		<0.001
Grade 0	59	39	20	21		7.0		13.0	
Grades 1–4	45	19	26	32		4.0		8.1	
Weight loss					<0.001		0.001		<0.001
Grade 0	49	39	10	13		6.5		11.2	
Grades 1–4	55	19	36	40		4.0		7.0	
Abdominal pain					0.043		0.034		0.006
Grade 0	91	54	37	45		6.5		11.0	
Grades 1–4	13	4	9	8		3.0		5.0	
Liver dysfunction					<0.001		<0.001		<0.001
Grade 0	68	44	14	29		7.0		12.1	
Grades 1–4	36	14	32	24		3.0		7.5	

*PD* progressive disease; *DCR* disease control rate; *TTP* time to progression; *OS* overall survival; *ECOG* Eastern Cooperative Oncology Group; *PS* performance status; *HBV* hepatitis B virus

### Response and Efficacy According to Intratumoral Microvessel Density

**Fig. 2:**
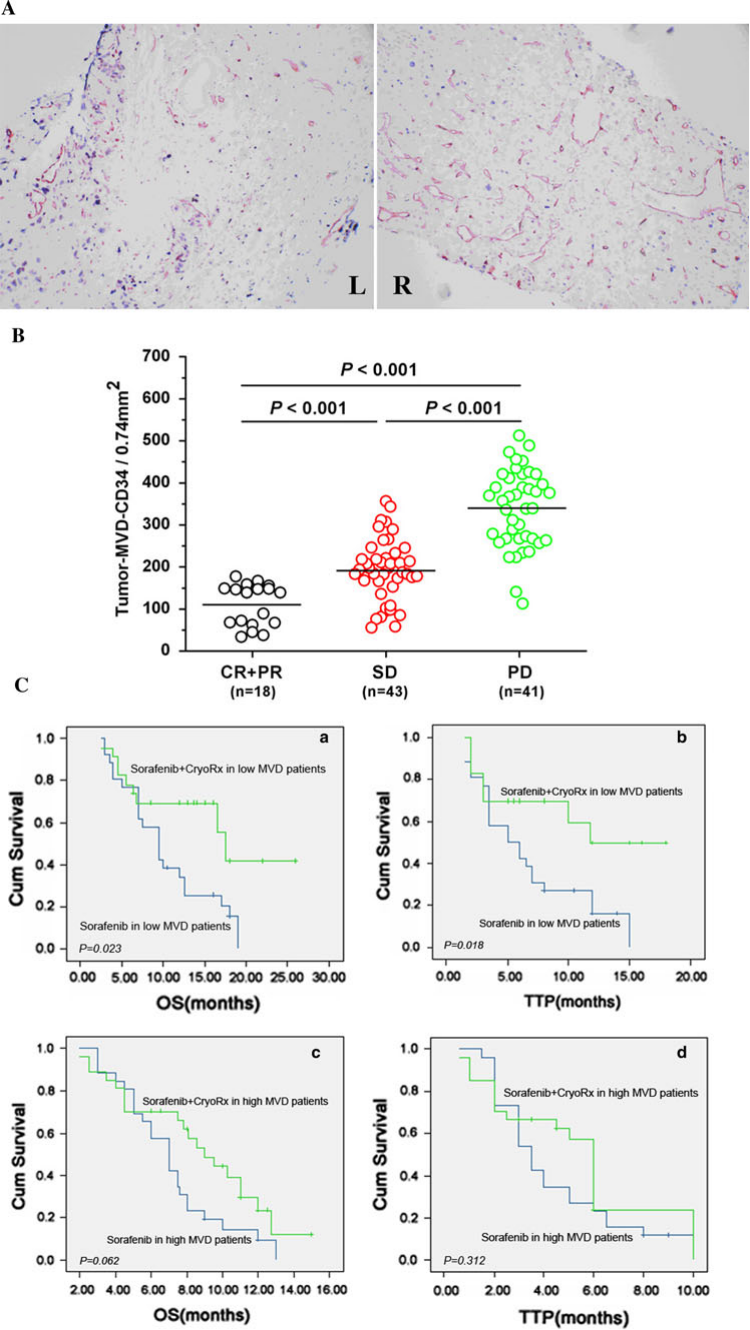
Comparison of intratumoral microvessels density (MVD)-CD34 in patients with advanced HCC and showing different overall responses.** A** Intratumoral microvessels density shown by anti-CD34 immunostaining (pink staining; *left* low MVD-CD34; *right* high MVD-CD34; ×200).** B** Mean intratumoral MVD-CD34 increased significantly (*P* < 0.001) with poor overall response.** C** A cohort of 104 patients was analyzed. Kaplan–Meier survival curves are shown for 52 patients with low MVD-CD34 (≤219.5/0.74 mm^2^). In this cohort, TTP (*a*) and OS (*b*) compared between patients from combination therapy group and sorafenib alone group differed significantly. Also, Kaplan–Meier survival curves are shown for 52 patients with high MVD-CD34 (>219.5/0.74 mm^2^). In this cohort, TTP (*c*) and OS (*d*) compared between patients from combination therapy group and sorafenib alone group differed significantly

Intratumoral microvessels density was observed by anti-CD34 immunostaining (×200; pink staining; left: low MVD-CD34; right: high MVD-CD34; Fig. 2A). Specific staining of capillary-like vessels was observed in all outcome groups (CR + PR: mean MVD-CD34, 111 ± 49/0.74 mm^2^, SD 206 ± 74/0.74 mm^2^, PD 339 ± 92/0.74 mm^2^; Fig. 2B). The mean MVD-CD34 (Fig. 2B) in the responsive (CR + PR) patients was significantly lower than that in PD patients (*P* < 0.001). At the time of analysis, the prognostic influence of MVD on the overall response and efficacy was evaluated by comparing OS or TTP between patients with low or high tumor MVD, determined by their median MVD value (median 219.5/0.74 mm^2^, ranging from 34 to 512/0.74 mm^2^). When the entire cohort of 104 patients was analyzed, 52 patients were found to show a lower than median MVD-CD34 (≤219.5/0.74 mm^2^). TTP and OS (Fig. 2C—a, b) differed significantly between combination therapy and sorafenib alone (log-rank: *P* = 0.018 for TTP; *P* = 0.023 for OS). In the 52 patients exhibiting a higher than median MVD-CD34 (>219.5/0.74 mm^2^), TTP and OS (Fig. 2C—c, d; Table 4) differed non-significantly between combination therapy and sorafenib alone (log-rank: *P* = 0.312 for TTP; *P* = 0.062 for OS).

**Table 4:** Univariate analysis of advanced HCC patients’ intratumoral MVD as a predictor of DCR, time to progressive, and OS

Parameter	No. of patients				*P*-value of DCR	TTP (months)		OS (months)	
	Total	DCR	PD	Died		Median 95 % CI	*P*	Median 95 % CI	*P*
Sorafenib + CryoRx					<0.001		0.007		0.006
MVD-CD34 low	25	23	2	7		11.0		17.5	
						8.5–14.5		15.2–19.8	
MVD-CD34 high	27	12	15	14		6.0		9.0	
						5.5–6.5		7.0–12.3	
Sorafenib					0.002		0.049		0.012
MVD-CD34 low	26	14	12	12		5.0		9.5	
						1.8–8.1		7.0–12.0	
MVD-CD34 high	26	9	17	20		3.5		6	
						2.6–4.4		4.6–8.2	
MVD-CD34 low					<0.001		0.018		0.023
Sorafenib + Cryo	25	23	2	7		11.5		17.5	
						8.8–14.9		15.2–19.8	
Sorafenib	26	14	12	12		5		9.5	
						2.0–8.1		7.0–12.0	
MVD-CD34 high					0.312		0.303		0.062
Sorafenib + Cryo	27	12	15	14		5		9	
						4.5–6.5		6.9–11.1	
Sorafenib	26	9	17	20		3.5		7	
						2.6–4.4		5.8–8.2	

*HCC* hepatocellular carcinoma; *MVD* microvessel density; *TTP* time to progression; *OS* overall survival; *DCR* disease control rate; *PD* progressive disease;* CryoRx* cryotherapy

### Continuation of Sorafenib in a Subset of Patients with Radiologic PD Improved OS

**Fig. 3:**
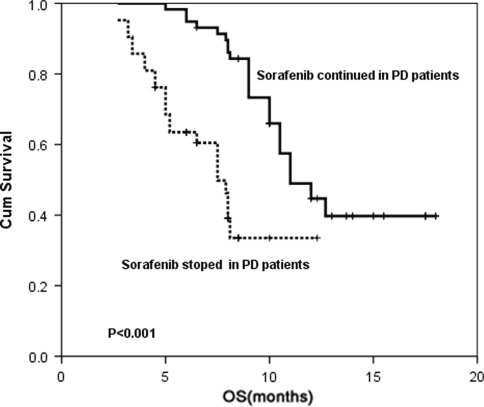
Kaplan–Meier analysis of the effect of continuing sorafenib therapy on OS in patients with radiologic PD. OS was significantly longer (*P* < 0.001) in PD patients from continuing sorafenib group than in PD patients from discontinued therapy group

At the end of follow-up, disease progression occurred in 86 patients. In 36 patients, sorafenib therapy was discontinued because of new lesions or concomitant clinical deterioration. However, 50 patients with a clinically stable presentation continued sorafenib therapy, despite disease progression. As shown in Fig. 3, OS was significantly longer (*P* < 0.001) in patients who continued sorafenib (11 months) as compared with those who discontinued therapy (7.5 months).

## Discussion

To our knowledge, this report is the first to describe that cryotherapy is associated with improved clinical outcomes in combination with sorafenib treatment for advanced HCC. Systemic chemotherapy has had a disappointing track record for treating advanced HCC [23]. However, sorafenib demonstrated a significant survival benefit and high tolerance in patients with advanced HCC in a phase III clinical trial [13]. Sorafenib was found to restrict tumor burden limit in advanced HCC [24]. It is important to reduce tumor burden to increase the clinical responses of drugs. Ablation therapies have been proposed as valid alternatives to surgery for the treatment of HCC in patients with cirrhosis [25]. A few studies have examined the outcomes of percutaneous cryoRx for HCC using CT monitoring and MR guidance, reporting that it was safe and effective [26, 27]. Moreover, it was found that not only was the local tumor necrotic, but the adjacent tumor tissue was also necrotic and shrunken in HCC patients following cryoRx, which was regarded as reflecting ectopic tumor suppression [28]. We reported that after cryoablation for small HCC the 1-, 2-, and 3-year recurrence-free survival rates were 72, 56, and 43 %, respectively [20]. As such, local recurrence after cryotherapy represents one of the main problems of this therapeutic strategy, and limits its associated survival benefits.

We speculated that combined sorafenib and cryoRx could be used to overcome tumor burdens and local recurrence in advanced HCC patients and provide significant survival benefits. Of note, in the present study, the median OS time of combination therapy patients was 12.5 months which was significantly longer than that of patients receiving sorafenib alone (8.6 months). In addition, the combination therapy significantly prolonged TTP and CER or DCR compared with sorafenib alone. The significant improvements in OS and TTP in the combination therapy provide encouraging evidence that the combination therapy may help overcome the tumor burden and local tumor recurrence. Indeed, the OS we report is longer than that reported in all previous studies on sorafenib treatment of advanced HCC patients [12, 13, 29–31]. All the patients in our study had advanced HCC (100 % of BCLC stage C and of HBV DNA positive) with macroscopic vascular invasion. In 50 % of our patients, the largest tumor diameter was >7 cm, and this characteristic suggests that the patients enrolled in our study might have more tumor burden than those enrolled in the previously reported trial [12, 13]. In the Asia–Pacific study [12], the median OS and TTP were 6.5 months and 2.8 months, respectively; and the population showed poorer performance (74 % ECOG PS ≥1) and a more advanced stage of cancer (96 % of BCLC stage C). In the SHARP study [13], the median OS and TTP were 10.7 months and 4.1 months, respectively; and the population exhibited a more advanced stage of cancer (82 % of BCLC stage C; 38 % macroscopic vascular invasion, and 51 % extrahepatic spread). The 12.5-month OS and 8.5-month TTP found in patients with PVT in the combination therapy are particularly impressive, in accordance with the rationale for the combination treatments by previous findings. Although we found that sorafenib could prolong survival in advanced HCC patients, monotherapy of sorafenib has not been found to produce tumor regression in HCC. Rather, high tumor load may render patients refractory to sorafenib [24]. In accordance with the above-referred evidence, our results suggest that the combination therapy has several advantages. First, the cryoRx can reduce the tumor burden to increase the efficacy of sorafenib. Second, sorafenib-mediated blockage of the Raf/MAPK and VEGFR pathways may enhance the efficacy of local cryoRx. Both these possibilities are supported by the present data. The clinical benefits of this treatment may be due largely to the reduction of tumor burden by cryoRx which corroborates the previous findings examining the effects of local ablation combined with TACE [32]. More importantly, addition of the cryoRx to sorafenib could further improve OS in these HCC patients; the profile, frequency, and degree of sorafenib-related adverse events (AEs) were comparable to previous reports and cryoRx did not further increase frequency and degree of sorafenib-related AEs. These encouraging results indicate that sorafenib combined with local treatment may provide the best therapeutic benefit in patients suffering from advanced HCC.

An important difference between our study and previous studies was the continuous administration of sorafenib, which may have also contributed to the survival benefit we observed. In the SHARP trial [12], a survival time of 5.2 months was reported after disease progression. In a Japanese phase I study of sorafenib [33], despite the median TTP being only 4.9 months, the median OS was relatively long (15.6 months). Yau et al. [24] reported that, even in patients who did not demonstrate any clinical benefits with sorafenib, OS was substantially better compared with their historical cohort. Wörns et al. [34] reported that radiologic disease stabilization (PR + SD) was achieved in 50 % of patients after a median of 3.2 months or at least a clinically stable presentation in a subset of patients with radiologic PD leading to continuation of therapy. These findings suggest that even the patients who exhibited no demonstrable clinical benefits with sorafenib treatment might obtain a survival benefit from the drug. Therefore, applying radiologic progression criteria would be likely to lead to the discontinuation of sorafenib therapy after 3–4 months in these cases, hence potentially denying these patients the opportunity to continue to receive clinical benefit and improved OS. We suggest that the decision to continue sorafenib therapy after radiologic progression is justified in patients with continuing clinically stable presentation.

Besides, sub-analyses were conducted on the basis of various factors associated with the prognosis of HCC patients that included age, the largest tumor diameter, tumor difference, ECOG PS, Child-Pugh class, and HBV DNA load. Our data show that sorafenib provided benefit to all the subpopulations analyzed, including those patients who normally show the worst outcomes. However, in patients with an ECOG PS of 2, those with the largest tumor diameter >7 cm, Child-Pugh class B, and high HBV DNA load, we also analyzed the correlation between treatment-related toxicities and prognosis. In corroboration to the previous findings [35], we found that fatigue, weight loss, and liver toxicity correlated, to some extent, with poor DCR, TTP, and OS. In another study [24], fatigue was observed in 50 % of patients. In the present study, 44 % of patients exhibited fatigue. We believe that severe fatigue may be a predictor of poor prognosis to a certain degree. Liver toxicity is an important issue during sorafenib treatment and local treatment. CryoRx can induce liver dysfunction/failure in HCC patients with stage C; however, addition of CryoRx to sorafenib can further improve the OS in these HCC patients. The profile, frequency, and degree of sorafenib-related AEs were comparable to the previous reports, and cryoRx did not further increase frequency and degree of sorafenib-related AEs. This finding suggests that the liver toxicity could be induced by sorafenib. Sorafenib can induce liver failure not only in Child-Pugh B patients but also in Child-Pugh A patients [36]. However, most sorafenib-induced liver failure, as we found, occurred in Child-Pugh B patients.

The previous studies [22, 37] reported intratumoral MVD as a prognostic measure of tumor. A prospective study [38] found a significant positive correlation between MVD and postoperative recurrence in patients undergoing resection of HCC ≤5 cm using CD34 as an endothelial cell marker. Therefore, we analyzed the correlation between MVD and response to sorafenib therapy. We found that mean MVD of patients with CER was significantly lower than that of patients with PD (*P* < 0.001). The results suggest that MVD affects the clinical response to therapy in advanced HCC patients. Among patients with low MVD, we found that those receiving the combination therapy exhibited a significantly longer median TTP and OS than those receiving sorafenib alone; but among patients with high MVD, TTP, and OS differed non-significantly between treatment groups. The current data suggest that the antiangiogenic effects of sorafenib for advanced HCC patients with a high MVD are mild. Since patients with complete PVT always have poor liver function (Child-Pugh class C), extrahepatic spread, and an expected survival time of less than 3 months, such patients were also excluded. Regarding the safety and efficacy of sorafenib combined with local cryoablation technique, further prospective, randomized, well-designed clinical studies will need to be taken up in the future.

In conclusion, this clinical study demonstrated that compared to sorafenib alone, the combined cryoRx and sorafenib therapy significantly improves TTP and OS in HBV-related BCLC stage C HCC patients with acceptable tolerance and similar safety profiles as previously reported. High intratumoral MVD was predictive of poor responses to sorafenib, and these results provide further validation for targeted therapy approach in advanced HCC patients.

## References

[1] Parkin DM (2001). Global cancer statistics in the year 2000. Lancet Oncology.

[2] Zhaoyou T (2009). Perspective of clinical oncology from the viewpoint of liver cancer studies. Tumor (China).

[3] Yoo HY, Patt CH, Geschwind JF (2003). The outcome of liver transplantation in patients with hepatocellular carcinoma in the United States between 1988 and 2001: 5-Year survival has improved significantly with time. Journal of Clinical Oncology.

[4] Yang HI, Lu SN, Liaw YF (2002). Hepatitis B e antigen and the risk of hepatocellular carcinoma. The New England Journal of Medicine.

[5] Sherman M (2005). Hepatocellular carcinoma: Epidemiology, risk factors, and screening. Seminars in Liver Disease.

[6] Llovet JM, Burroughs A, Bruix J (2003). Hepatocellular carcinoma. Lancet.

[7] Llovet JM, Burroughs A, Bruix J (1999). Prognosis of hepatocellular carcinoma: The BCLC staging classification. Seminars in Liver Disease.

[8] Bruix J, Llovet JM (2002). Prognosis prediction and treatment strategy in hepatocellular carcinoma. Hepatology.

[9] Bruix J, Sherman M (2005). Management of hepatocellular carcinoma. Hepatology.

[10] Park KW, Park JW (2008). Survival analysis of 904 patients with hepatocellular carcinoma in a hepatitis B virus-endemic area. Journal of Gastroenterology and Hepatology.

[11] Llovet JM, Fuster J, Bruix J, Barcelona-Clinic Liver Cancer Group (2004). The Barcelona approach: Diagnosis, staging, and treatment of hepatocellular carcinoma. Liver Transplantation.

[12] Cheng AL, Kang YK, Chen Z (2009). Randomized phase III trial of sorafenib versus placebo in Asian patients with advanced hepatocellular carcinoma. Lancet Oncology.

[13] Llovet JM, Ricci S, Mazzaferro V, SHARP Investigators Study Group (2008). Sorafenib in advanced hepatocellular carcinoma. The New England Journal of Medicine.

[14] Furuse J (2008). Sorafenib for the treatment of unresectable hepatocellular carcinoma. Biologics.

[15] Hakimé A, Hines-Peralta A, Peddi H (2007). Combination of radiofrequency ablation with antiangiogenic therapy for tumor ablation efficacy: Study in mice. Radiology.

[16] Atwell TD, Farrell MA, Callstrom MR (2007). Percutaneous cryoablation of 40 solid renal tumors with US guidance and CT monitoring: Initial experience. Radiology.

[17] Jungraithmayr W, Burger D, Olschewski M, Eggstein S (2005). Cryoablation of malignant liver tumor: Results of a single center study. Hepatobiliary & Pancreatic Diseases International.

[18] Lencioni R, Llovet JM (2010). Modified RECIST (mRECIST) assessment for hepatocellular carcinoma. Seminars in Liver Diseases.

[19] Trotti A, Colevas AD, Setser A (2003). CTCAE v3.0: Development of a comprehensive grading system for the adverse effects of cancer treatment. Seminars in Radiation Oncology.

[20] Wang C, Lu Y, Chen Y (2009). Prognostic factors and recurrence of hepatitis B-related hepatocellular carcinoma after argon–helium cryoablation: A prospective study. Clinical Experimental Metastasis.

[21] Kuang DM, Wu Y, Chen N, Cheng J, Zhuang SM, Zheng L (2007). Tumor-derived hyaluronan induces formation of immunosuppressive macrophages through transient early activation of monocytes. Blood.

[22] Poon RT, Ng IO, Lau C (2002). Tumor microvessel density as a predictor recurrence after resection of hepatocellular carcinoma: A prospective study. Journal of Clinical Oncology.

[23] Yau T, Chan P, Epstain R, Poon RTP (2009). Management of advanced hepatocellular carcinoma in era of target therapy. Liver International.

[24] Yau T, Chan P, Ng KK (2009). Phase 2 open-label study of single-agent sorafenib in treating advanced hepatocellular carcinoma in a hepatitis B-endemic Asian population: Presence of lung metastasis predicts poor response. Cancer.

[25] Greten TF, Korangy F, Manns MP, Malek NP (2009). Molecular therapy for the treatment of hepatocellular carcinoma. British Journal of Cancer.

[26] Zuro, L. M., & Staren, E. D. (1996). Cryosurgical ablation of unresectable hepatic tumors. *Association of perioperative Registered Nurses (AORN) Journal, 64*, 231–236, 239–244. 10.1016/s0001-2092(06)63151-7 8853781

[27] Shimizu T, Sakuhara Y, Abo D (2009). Outcome of MR-guided percutaneous cryoablation for hepatocellular carcinoma. Journal of Hepatobiliary & Pancreatic Surgery.

[28] Osada S, Imai H, Tomita H (2007). Serum cytokine levels in response to hepatic cryoablation. Journal of Surgical Oncology.

[29] Shim JH, Park JW, Choi JI, Park BJ, Kim CM (2009). Practical efficacy of sorafenib monotherapy for advanced hepatocellular carcinoma patients in a hepatitis B virus-endemic area. Journal of Cancer Research and Clinical Oncology.

[30] Pinter M, Sieghart W, Ivo Graziadei I (2009). Sorafenib in unresectable hepatocellular carcinoma from mild to advanced stage liver cirrhosis. The Oncologist.

[31] Kane RC, Farrell AT, Madabushi R (2009). Sorafenib for the treatment of unresectable hepatocellular carcinoma. The Oncologist.

[32] Cheng BQ, Jia CQ, Liu CT (2008). Chemoembolization combined with radiofrequency ablation for patients with hepatocellular carcinoma larger than 3 cm: A randomized controlled trial. Journal of the American Medical Association.

[33] Furuse J, Ishii H, Nakachi K (2008). Phase I study of sorafenib in Japanese patients with hepatocellular carcinoma. Cancer Science.

[34] Wörns MA, Weinmann A, Pfingst K (2009). Safety and efficacy of sorafenib in patients with advanced hepatocellular carcinoma in consideration of concomitant stage of liver cirrhosis. Journal of Clinical Gastroenterology.

[35] Vincenzi B, Santini D, Russo A (2010). Early skin toxicity as a predictive factor for tumor control in hepatocellular carcinoma patients treated with sorafenib. Oncologist.

[36] Schramm C, Schuch G, Lohse AW (2008). Sorafenib-induced liver failure. American Journal of Gastroenterology.

[37] Semela D, Dufour JF (2004). Angiogenesis and hepatocellular carcinoma. Journal of Hepatology.

[38] Liu L, Cao Y, Chen C (2006). Sorafenib blocks the RAF/MEK/ERK pathway, inhibits tumor angiogenesis, and induces tumor cell apoptosis in hepatocellular carcinoma model PLC/PRF/5. Cancer Research.

